# Design of a Superhydrophobic Photothermal Shape-Memory Material Based on Carbon-Nanotubes-Doped Resin for Anti-Icing/De-Icing Applications

**DOI:** 10.3390/ma18112540

**Published:** 2025-05-28

**Authors:** Yingcheng Zhao, Pei Tian, Xinlin Li, Di Gai, Wei Tong

**Affiliations:** 1 School of Astronautics, Harbin Institute of Technology, Harbin 150080, China; zhaoyingc2024@163.com (Y.Z.); 24s118136@stu.hit.cn (P.T.); 2 Centre for Composite Materials and Structures, Harbin Institute of Technology, Harbin 150080, China; 3 School of Safety Engineering, Shenyang Aerospace University, Shenyang 110136, China; tomson90@126.com; 4 School of Mechanical and Aerospace Engineering, Nanyang Technological University, Singapore 639798, Singapore

**Keywords:** shape-memory polymer, photothermal effect, superhydrophobicity, anti-icing, de-icing

## Abstract

Icing on power lines and wings can cause serious economic damage and safety hazards. While superhydrophobic materials show promise for anti-icing applications, their passive anti-icing mechanisms require external energy activation, highlighting the need for the development of active de-icing materials with energy-to-heat conversion capabilities. Here, we developed three photothermal superhydrophobic shape-memory polymers with anti-icing performance (PSSPs), with 3%, 5%, and 7% CNT doping ratios, through a two-step process: resin preparation and laser-processing modification. The results showed that all samples presented good superhydrophobic properties. In addition, the tested materials demonstrated good shape-memory performance (recovery rates were close to 100%). They also showed excellent de-icing performance. Owing to the simplicity of the fabrication process, the material is suitable for mass production. The synergistic interplay between superhydrophobicity and photothermal activation endows the material with dual-functional icephobic performance, demonstrating practical applicability in industrial cryogenic environments.

## 1. Introduction

Ice accretion poses a safety risk in various modes of transportation, including aircraft and high-speed rail, in addition to ship decks, radar, offshore oil platforms, and other infrastructure and systems. The phenomenon of icing can create significant hazards that must be addressed [1,2,3,4,5,6,7]. Additionally, this phenomenon is harmful in many scenarios: the icing of aircraft wings can cause deterioration in flight performance, the icing of railway catenary lines can cause damage to pantographs, and the icing of cooling towers can lead to poor cooling. Current de-icing strategies, including manual ice removal, mechanical de-icing, and heating to melt ice, demonstrate limited efficacy in icing prevention. Though widely adopted, these traditional measures induce structural fatigue risks while introducing unnecessary complexity in systems. Therefore, there is an urgent need for a de-icing method, one based on the properties of the material itself, to complement these methods.

In nature, the surfaces of the plants denoted as colocasia have superhydrophobic and self-cleaning properties. Inspired by these functions of colocasia, we can design anti-/de-icing surfaces to minimize water-droplet retention by leveraging trapped air layers, which reduces thermal conductivity and effectively delays ice formation. Some studies have shown that superhydrophobic surfaces exhibit icephobic properties in specific environments. This enhanced droplet mobility facilitates rapid shedding while suppressing heterogeneous ice nucleation by controlling energy dissipation at the solid–liquid interfaces, and enhances droplet rebound dynamics by minimizing interfacial contact duration [8,9]. Zhang et al. achieved an average water contact angle (WCA) of 163.0° by etching on 304 stainless steel and coating TiN nanoparticles after in situ oxidation [10]. Xuan et al. prepared a material, the WCA of which was 162°, by using a femtosecond laser modified with 1H, 1H, 2H, 2H-perfluorodecyltriethoxysilane (PFTS) [11]. Conventionally, bioinspired superhydrophobic surfaces have faced a threefold implementation barrier: insufficient cryogenic durability, absence of active ice-repellent functionality, and substrate versatility limitations. These critical shortcomings impede practical implementations in dynamic thermal environments. The interfacial ice phase cannot be effectively separated without energy-assisted intervention measures. Energy in the form of heat could be supplied to the material to melt the ice and achieve de-icing. An anti-icing material with photothermal functions can keep the temperature of the material itself above the freezing point to prevent icing. Li et al. achieved the functionalization and uniform dispersion of CNT by grafting superhydrophobic silica onto the surface of CNT, and the prepared 15% phase-change superhydrophobic photothermal CNT content coating was heated from −10 °C to 50 °C in 400 s under 1 kW/m^2^ light, demonstrating good photothermal properties [12]. Wei et al. prepared a coating by spraying a dispersion composed of fluorinated silica nanoparticles, silicon-modified polyester binders, and photothermal carbon-black nanoparticles onto Al alloy plates, and the surface temperature of the as-prepared photothermal materials increased rapidly from −18.3 °C to 0 °C after 115 s irradiation under low solar flux (0.2 sun) [13].

Significant research efforts have focused on stimuli-responsive surfaces incorporating shape-memory polymeric composites, in which programmable microstructural geometries can be dynamically reconfigured through external triggers [14,15]. Shape-memory polymers (SMPs) stand out among intelligent material systems due to their ability to memorize and recover predetermined configurations under specific environmental cues [16,17,18]. The temporary morphologies of SMPs can be thermally/optically encoded and subsequently reverted to their original states through stimuli-responsive phase transitions triggered by thermal, optical, or electromagnetic inputs [19,20,21,22]. These polymeric materials exhibit a suite of advantageous characteristics, including adjustable rigidity, controllable shape morphing, compatibility with composite integration, high strain tolerance, and ease of processing [23]. The integration of SMPs into icephobic material systems diversifies their application spectrum, enhancing adaptability across complex operational environments. Shape-memory polymers (SMPs) enable reconfigurable hydrophobic architectures through programmable stiffness modulation. Superhydrophobic materials with variable shapes are also advantageous in water transportation [24,25,26,27].

Cheng’s group pioneered interfacial architecture fabrication techniques, enabling real-time wettability transitions between uniform and directional wetting states. These compressed cylindrical arrays can change the direction of the droplets or directly fix the droplets and can be returned to their original state by heating to 100 °C [28]. Hao et al. synthesized a carbon-black/epoxy composite exhibiting integrated superhydrophobicity, photothermal conversion, and light-activated shape-memory behavior, achieving near-complete shape recovery under optical stimulation, and showing an icing delay of up to 337 s at ≥−25 °C [29]. Bai et al. designed ordered microtrench architectures that were laser-patterned on SMP surfaces using femtosecond laser-processing, enabling dynamically tunable anisotropic wettability through shape-memory effects; in these patterns, the anisotropic wettability was modulated by varying the ridge width (RW) and groove depth (DG) of the microgroove array [30].

In this study, a photothermal superhydrophobic shape-memory polymer (PSSP) with anti-/de-icing performance and based on shape-memory composites was prepared, and its wettability was studied by measuring its water contact angle (WCA) and water sliding angle (WSA). The material was systematically characterized as to its hydrophobicity, mechanical behavior, and thermal stability. Thermomechanical cycling was systematically implemented to assess the shape-memory characteristics, with particular emphasis on shape recovery ratio and cyclic durability, including recovery efficiency and repeatability. Fourier-transform infrared spectroscopy (FTIR) confirmed the successful chemical modification of the composite’s surface. The integrated anti-icing resistance and photothermally activated de-icing functionality were systematically evaluated under standardized photic control, demonstrating delayed ice nucleation and accelerated ice removal. The synergistic combination of photothermal response, mechanical durability, and dual anti-icing/de-icing functions positions this material as a promising candidate for practical ice-mitigation applications.

## 2. Materials and Methods

### 2.1. Materials

Carbon nanotubes (CNT) and bisphenol-A-type epoxy resin were purchased from Suzhou Tanfeng Graphene Technology Co., Ltd. (Suzhou, China) and Nantong Xingchen Synthetic Material Co. Ltd. (Nantong, China), respectively. Shape-memory epoxy resin (SMEP) was made, using bisphenol-A epoxy resin and Jeffamine D230, in our lab. 1H,1H,2H,2H-Perfluorooctyltriethoxysilane (PFOTES) was provided by Aladdin reagent (Shanghai, China). Jeffamine D230 and ethyl acetate were provided by Macklin Biochemical Co., Ltd. (Shanghai, China).

### 2.2. Sample Preparation

#### 2.2.1. Preparation of Photothermal Epoxy Substrates

Carbon nanotubes were added to the laboratory-synthesized shape-memory resin, according to weight fractions of 3%, 5%, and 7%, respectively; 10 mL of ethyl acetate was then added to each sample, and this was followed by magnetic stirring for 2 h. The composite suspension was transferred into a PTFE cavity and subjected to thermal processing involving initial stabilization (80 °C × 2 h) followed by final crosslinking (100 °C × 1 h).

#### 2.2.2. Surface Engineering of CNT/Shape-Memory Epoxy Composites via Laser

The microstructures were developed on the surface by laser; the line spacing was adjusted to 0.01 mm, the speed was 500 mm/s, the processing power was 50%, and the number of processing cycles is 2.. The processed sample was put into a beaker filled with ethanol, in which it was ultrasonically cleaned for 15 min. Then, the sample was dried and put into a 5 wt% PFOTES ethanol solution for 2 h for modification and then dried at 80 °C for 2 h to obtain the superhydrophobic shape-memory materials.

### 2.3. Testing and Characterization

#### 2.3.1. Characterization

Topographic evaluation was performed with the SU5000 SEM (HITACHI, Tokyo, Japan) equipment, INVENIO S spectrometer (Bruker, Ettlingen, German) used for infrared analysis and an AXISULTRA DLD (Kratos, Stretford, UK) system used for elemental surface characterization. The surface wettability of the superhydrophobic and smooth coatings was characterized at ambient temperatures, using a contact angle meter (DSA 25S, Krüss, Hamburg, German), with 5 μL water droplets.

#### 2.3.2. Shape-Memory Behavioral Representations

Thermomechanical property assessment was carried out using dynamic mechanical analysis (DMA, Q800, TA Instruments, New Castle, DE, USA) under the strain mode, subjecting specimens to temperature progressions (25–200 °C, 3 °C/min) and periodic stress at 1 Hz. The shape-memory functionality of the PSSP specimen (10 × 30 mm) was quantified through recovery rate measurements and temporal response profiling. The actuation behavior of the U-shaped PSSP was monitored upon thermal stimulation (>70 °C), capturing the complete recovery trajectory from deformed state to permanent geometry.

#### 2.3.3. Characterization of Icephobic Efficacy

The anti-icing performance was studied outdoors (Shuangyashan, China) at an outdoor temperature of −20 °C; 50 μL aqueous droplets were deposited onto cryogenically maintained substrates. The cryogenic phase transition was temporally analyzed through solidification latency measurements.

#### 2.3.4. Photothermal Performance Investigation

A 50 μL deionized water droplet was deposited on the sample’s surface and cryofixed in subzero conditions. Following icing, the surface was irradiated with 808 nm near-infrared (NIR) illumination at 1 W power output. Real-time temperature evolution within the ice layer was monitored via embedded thermocouples. Three replicate specimens underwent five consecutive 60 s photothermal cycles under identical NIR exposure parameters.

## 3. Results and Discussion

### 3.1. Shape-Memory Performance

**Figure 1:**
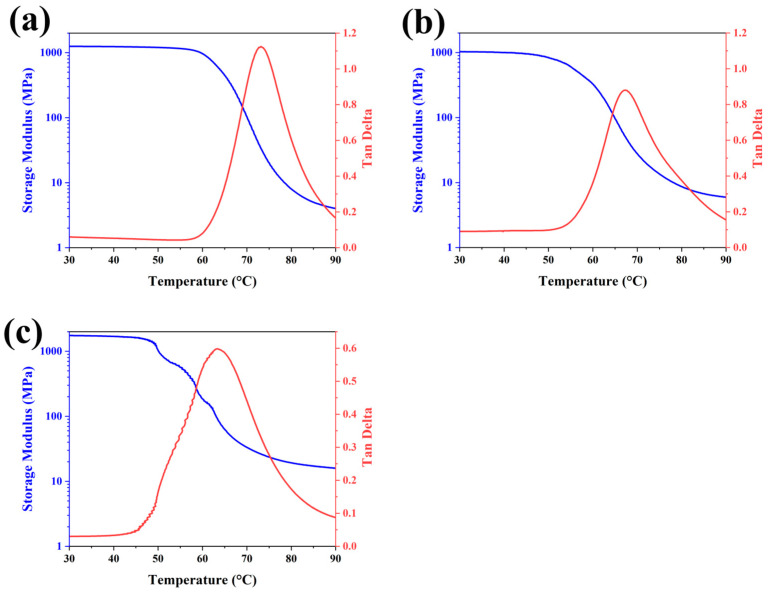
DMA images of PSSPs with three different CNT doping ratios. (**a**), (**b**), and (**c**) represent figures with rates of 3%, 5%, and 7%, respectively.

Shape-memory materials enable active deformation upon thermal stimulation, effectively breaking ice adhesion through the means of controlled structural changes, which enhances de-icing efficiency without external mechanical intervention. The temperature-dependent viscoelastic transitions characteristic of SMPs were quantitatively profiled through dynamic mechanical analysis (DMA) measurements, enabling accurate identification of the glass transition temperature (T_g_). Figure 1 illustrates the temperature-dependent storage modulus (E′) profiles obtained from dynamic mechanical analyses of all distinct samples. The observed E′ decays exponentially with increasing thermal excitation, suggesting that the material is gradually softening as the temperature rises. DMA revealed a marginally elevated initial storage modulus (E′) in the 7% formulation, compared to its 3% and 5% counterparts. During thermal ramping, the 3% and 5% groups exhibited marked E′ reduction (~65 °C), whereas the 7% system demonstrated stepwise E′ decline, with subsequent stabilization at this thermal transition threshold. Upon reaching 90 °C, the three specimens exhibited sequentially enhanced storage moduli, with its progression demonstrating direct proportionality to the CNT doping concentrations, thereby confirming the dopant-dependent stiffening effect. The peak of the loss factor (Tan δ) revealed T_g_ values of 73.2 °C, 67.19 °C, and 63.6 °C for the three samples, respectively, with the T_g_ reduction (60–70 °C range) inversely correlated to CNT doping ratios in the photothermal superhydrophobic shape-memory polymer with anti-icing (PSSP) matrix. Observing the peak value of Tan δ, it was found that it decreased with the increase of CNT doping concentration, which indicated that the CNT doping concentration had a negative correlation effect with respect to Tan δ.

**Figure 2:**
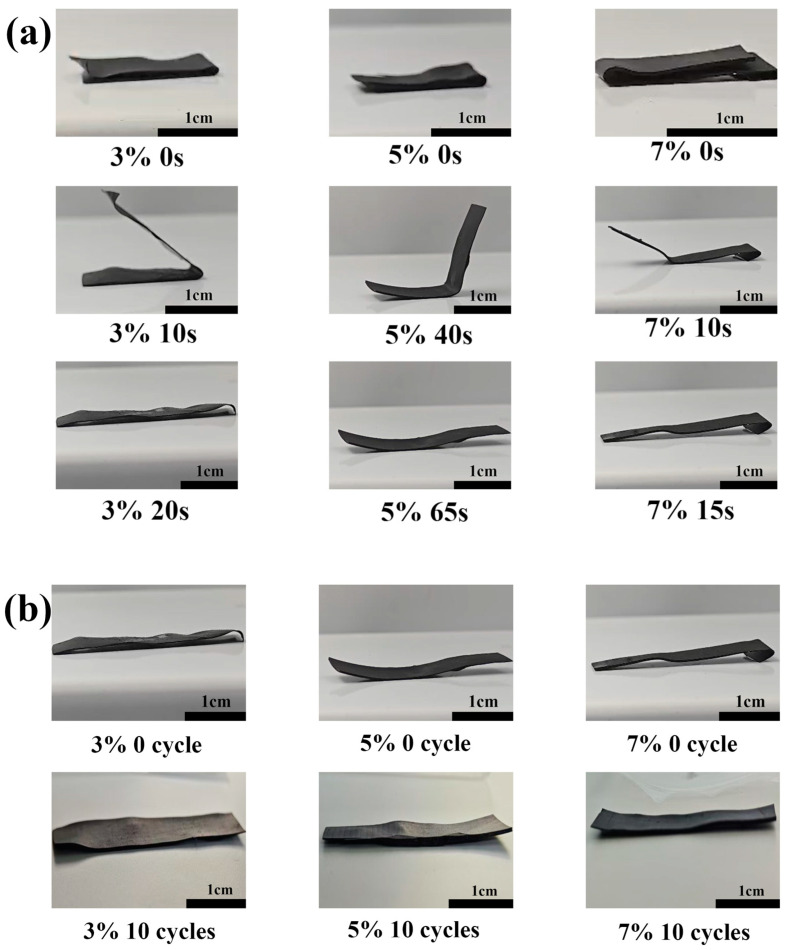
(**a**) The deformation of 3%, 5%, and 7% CNT-doped PSSPs over time in one heating and cooling cycle, and (**b**) the shape change of each sample before and after 10 heating reformation cycles; the sample is in a completely cooled state.

The shape-memory effect is activated through a four-stage thermomechanical conditioning protocol initiated by thermal excitation beyond the T_g_, followed by stress-induced deformation, vitrificated change, and final recovery once heated above the T_g_. In the experiment, we heated the samples with the three doping ratios to 70 °C, a temperature above T_g_, bent them into a “U” shape (0 s state) and cooled them. We then placed them on a laboratory bench heated to 70 °C for shape restoration experiments, and recorded videos to observe the relationship between their recovery state and time. As shown in Figure 2a, the 3% sample spontaneously bent 45° after 10 s and fully returned to its initial state after 20 s. The total recovery time of the 7% sample was close to that of the 3% sample, only 15 s, and the spontaneous bending at 10 s had reached 150°, exhibiting substantially enhanced activation kinetics relative to the other two samples. The 5% sample showed a spontaneous bending of 100° at 40 s and returned to its original state at 65 s. Overall, all three samples had a fast activation rate and could quickly return to their original states. This is essential for the cracking of the ice on the surface of the material, as it means that the ice is removed by physical fracture rather than phase change, which significantly saves de-icing time and reduces heat loss. After the feeding of the three samples into a 70 °C oven to heat them to T_g_, the samples were taken out for bending and fixing, cooled and formed, and then put into the oven for reformation after being completely cooled; they had completed a cycle after they were fully extended. Ten cycles of experiments were performed on the three samples. As shown in Figure 2b, the material reformation rate after ten cycles reaches 100%. It is critical to determine the shape-recovery performance of the PSSP after multiple cycles, as this determines the service life of the material, and a long-life material means lower costs and a lower replacement frequency. The process of this experiment is illustrated in Video S1–S3.

### 3.2. Physicochemical Analysis of CNT/Shape-Memory Epoxy Composites

Integrated with superhydrophobic surfaces, shape-memory composites synergistically delay ice formation and facilitate ice detachment via surface morphology adjustments.

**Figure 3:**
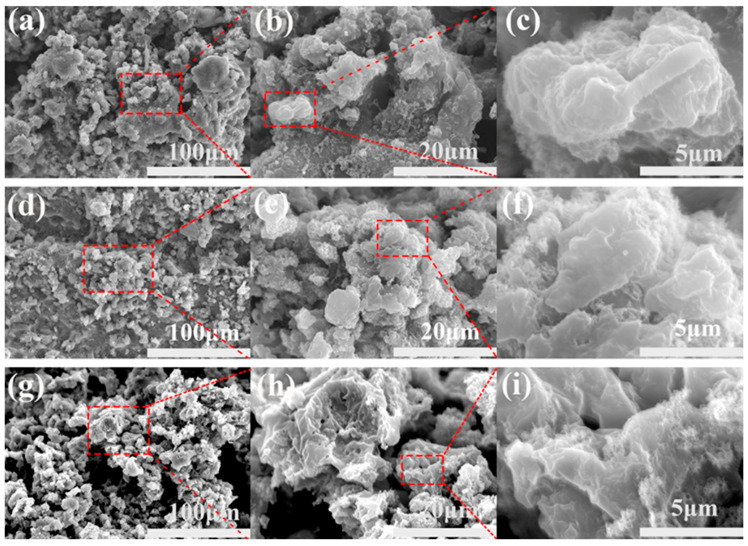
Scanning electron micrographs (**a**–**i**) systematically characterize the surface morphology of CNT-doped PSSP composites at varied doping levels (3%, 5%, and 7%) and at different magnifications. Specifically: (**a**–**c**) 3% CNT loading, (**d**–**f**) 5% CNT loading, and (**g**–**i**) 7% CNT loading.

Figure 3 shows SEM images of three samples at different magnifications; Figure 3a–c show SEM images of 3% CNT-doped PSSP. Microstructural analysis revealed a substantial population of microscale protrusions and interconnected troughs across the specimen surface and demonstrated marked alterations in surface topography following laser irradiation. The specific morphologies and surface structures of these micron-scale protrusions and gullies can be observed in enlarged Figure 3b,c. Figure 3d–f show SEM images of 5% CNT-doped PSSP, from which it can be seen that the particles formed by 5% CNT-doped PSSP are smaller and denser, and the gullies are shallower than those of the 3% doped product; this is better demonstrated in the enlarged images of Figure 3e,f. The 7% CNT-doped PSSP shown in Figure 3g–i exhibited three-dimensional structures of higher complexity that may not contribute to light absorption; this may be due to the increase in CNT content. In the enlarged images of Figure 3h,i, finer but deeper gullies are found, suggesting that there are some complex structures under the photographed structure. These complex surface structures contribute to the excellent hydrophobic properties of the material.

**Figure 4:**
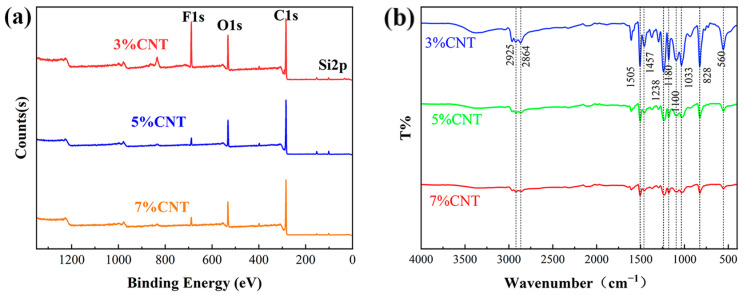
(**a**) XPS spectra of 3%, 5%, and 7% CNT-doped PSSPs; (**b**) FTIR spectral images of 3%, 5%, and 7% CNT-doped PSSPs.

Surface chemical composition, in particular, low surface-energy coatings, was important in synergistically enhancing superhydrophobicity. In Figure 4a, XPS measurements show the photoionization peaks for carbon, oxygen, silicon, and fluorine in the CNT-doped PSSP measurement spectra, and it can be observed that all three ratios of the sample contain F1s and Si2p peaks representing modification through grafting. At the same time, the fluorine content of the 3% CNT sample was higher than those of the other two, indicating that more fluorine-containing hydrophobic chains were grafted in the 3% doped sample than in the latter two. This may be due to the fact that the 3% CNT-doped resin contains less of the tested doping than do the 5% and 7% CNT, which makes the CNT network inside the material poor, resulting in the poor thermal conductivity of the material, and the laser energy absorbed on the surface cannot be quickly conducted to the bottom; eventually, the material closer to the surface is ablated, revealing more epoxy resin components. These epoxy resin components are more conducive to the adsorption of PFOTES. The confirmation of the presence of F and Si is helpful for confirming the results of grafting.

Figure 4b shows the FITR spectra of 3%, 5%, and 7% CNT-doped PSSP. The 2925 and 2864 cm^−1^ wavenumber peaks in Figure 4b indicate the symmetrical and anisotropic electron density distribution along the bond axis modes of the C-H bond designated as CH, respectively, and 1467 cm^−1^ may be C-O from epoxy resin [31,32]. The peaks at 1100 and 828 cm^−1^ indicate the axial deformation modes of Si-O-C (methoxy) and Si-C (alkyl) bonds, which exhibit distinct shifts; 1238 cm^−1^ is a C-C bond; 1505 cm^−1^ is a benzene ring skeleton vibration; 828 and 560 cm^−1^ correspond to the antisymmetric and symmetrical tensile vibration peaks of Si-O-Si, respectively; and the C-F bond presents asymmetric and symmetrical axial deformation bands at 1238 cm^−1^ and 1180 cm^−1^, respectively, belonging to the CF_2_ and CF_3_ groups [33]. The concomitant presence of fluorine and silicon X-ray photoelectron spectroscopy (XPS) signatures confirms the covalent grafting of 1H,1H,2H,2H-Perfluorooctyltriethoxysilane (PFOTES) molecular chains onto the substrate surface, and the hydrophobicity of the micro–nano structure brought by the chain-like side branches in the molecule has been obtained.

### 3.3. Self-Cleaning Properties

**Figure 5:**
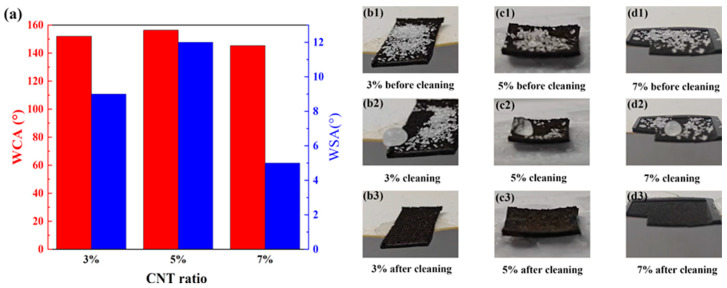
(**a**) Water contact angle (WSA) and water contact angle (WCA) for the three samples; (**b1**–**d3**) self-cleaning effects of the three different proportions of CNT-doped PSSP.

In the real world, where the material may become dusty and degrade, the material’s self-cleaning ability is important. The superhydrophobic aspects and self-cleaning performance of the material were tested by cleaning the dust from its surface. From Figure 5a, it can be determined that the WCA of all three groups is above 145°, and the WSAs of the 3% and 7% groups are below 10°, showing good superhydrophobic properties; this is conducive to normal performance in anti-icing and the functioning of self-cleaning properties. The self-cleaning test was carried out on three samples, as shown in Figure 5(b1,c1,d1). Quartz sand particles were deposited onto the substrate surface to simulate particulate contamination; subsequently, a 50 μL water-droplet rinsing effectively removed the adhered pollutants through the self-cleaning action inherent in the superhydrophobic interface, so the three samples showed good self-cleaning performance, one which was suitable for maintaining their superhydrophobic and photothermal anti-icing capabilities. The process of this experiment is illustrated in Video S4–S6.

Interestingly, it can be seen from the three diagrams of b2, c2, and d2 in Figure 5 that the water droplets do not simply wash away the quartz sand, but fall from the edge together with the quartz sand. This phenomenon corroborates the material’s inherent low surface-energy properties. The binding energy of the water and the quartz sand is greater than the binding energy of either of the two to the surface of the material.

### 3.4. Anti-/De-Icing Properties

For icephobic material systems, the dual-functional capacity of preventing ice accretion and facilitating ice removal constitutes a critical functional parameter requiring comprehensive evaluation under a standardized process.

The freezing mechanism of surface-adhered droplets progresses through sequential thermal phases: an initial metastable supercooling period in which liquid persists below its crystallization point, followed by an abrupt phase-transition stage initiated by nucleation events that release latent heat, and ultimately complete solidification is achieved through interfacial heat transfer governed by droplet geometry and environmental conditions.

$$t=\frac{\rho_{l}LV}{hS(T_{m}-T_{w})}$$

Therein, $\rho_{l}$ is the density of water, L is the latent heat of phase change, V is the droplet volume, h is the heat transfer coefficient, S is the contact area, $T_{m}$ is the equilibrium freezing temperature, and $T_{w}$ It is the surface temperature [34].

**Figure 6:**
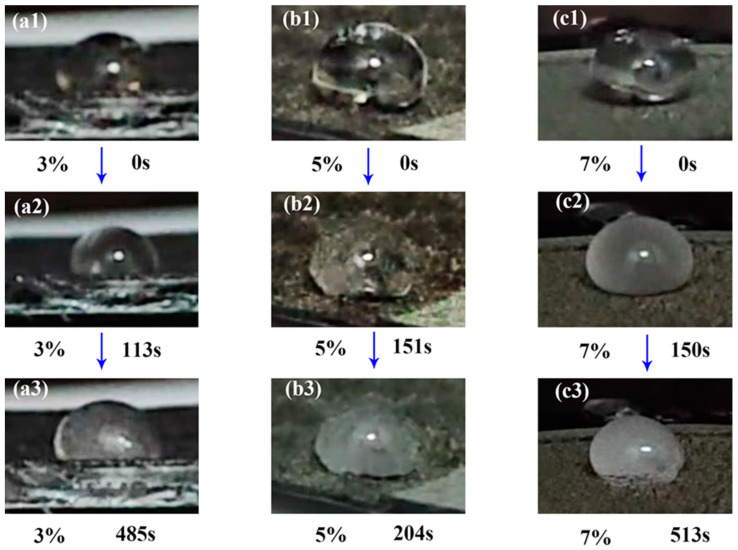
Icing time on the surfaces of the 3%, 5%, and 7% CNT-doped shape-memory resins. (**a1**–**a3**), (**b1**–**b3**), and (**c1**–**c3**) represent the figures of the 3% sample, 5% sample, and 7% sample, respectively.

As depicted in Figure 6, under subzero ambient conditions (−20 °C), water droplets deposited on the PSSP’s surface maintain optical transparency at the onset of exposure (t = 0 s) and become non-transparent after becoming completely frozen. The initial icing times of the three samples are 113 s, 151 s, and 150 s. The process of this experiment is illustrated in Video S7–S9.

**Figure 7:**
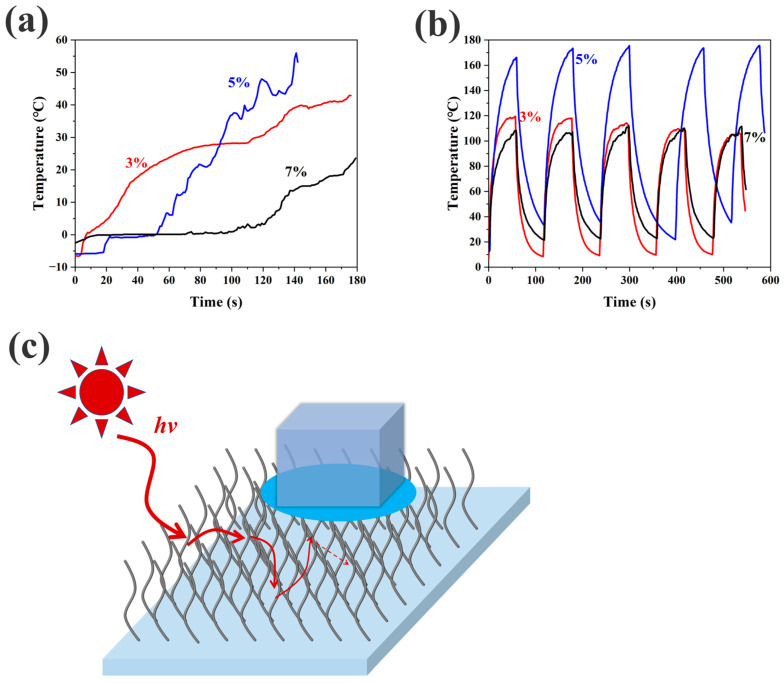
(**a**) The temperature curves for the ice of the three samples in the photothermal ice-melting experiment. (**b**) The photothermal cycling-induced surface thermal signatures exhibited distinct temporal evolution patterns among the three PSSP formulations. (**c**) A schematic diagram of the principle of CNT-enabled thermo-optical energy transduction—microchannel-textured substrates for enhanced photon-trapping.

In the interglacial temperature curves shown in Figure 7a, it can be seen that since the location of the thermocouple is also affected by the surface temperature of the PSSP, the materials can heat up to temperatures that would be impossible to reach inside a surface ice particle, which is very confusing and could be mistaken for the ice itself melting rapidly. So, the ice melt period is determined by observing the “plateau” of the temperature. Comparative analyses of phase-stabilization durations revealed distinct concentration-dependent behavior: The 5 wt% formulation exhibited a transient equilibrium phase (~30 s), whereas the 3 wt% and 7 wt% systems demonstrated prolonged stabilization intervals approximating 80 s and 100 s, respectively; this is also evidenced by the fact that the 5% samples rise to a higher temperature during the subsequent heating process in the ice/water scenario. The 5% CNT-doped PSSP samples exhibit outstanding photothermal properties. As shown in Figure 7b, this is also proved in the temperature curves of the photothermal cycles, and the temperature peaks of the three samples within 1 min of illumination are stable in 5 cycles. In the later stages, the samples’ temperature peaks in both the 3% and 7% groups tended to be 120 °C, while the sample in the 5% group performed outstandingly, reaching a high temperature of nearly 180 °C and performing consistently over several cycles. The process of these two experiments is shown in support information and Figure S1.

Interestingly, the 5% CNT-doped material did not perform well in the anti-icing experiment, but did perform well in the de-icing experiment. This may be due to the fact that the 5% has a deeper trough than the 3% and fewer voids than the 7%, which means less air in the voids, which, in turn, means worse hydrophobic and anti-icing properties, which, in turn, may mean that PSSPs with different CNT doping ratios can be more beneficial in different scenarios. To explain this phenomenon, it is necessary to return to the essence of the photothermal properties of the PSSP materials and further analyze their photothermal mechanism. The photothermal performance of CNT-doped materials comes from its light absorption, specifically the properties of light capture on the surface of the material, and to achieve high photothermal performance, enhancement of photon harvesting efficiency at the material interface is imperative, as experimentally validated through spatially resolved optoelectronic characterization. In Figure 7c, on the one hand, the capture rate of light depends on the amount of CNT doped in the material, and the material affected by CNT doping turns from transparent to black, which improves the absorbance of the material. In addition, CNT is an sp^2^ hybrid material; it can absorb photons to acquire the electrons in their excited state, and return them to the ground state in a variety of ways to release energy, and part of the released energy is reflected in thermal energy, as a part of the range of photothermal energy. In addition, CNTs have excellent thermal conductivity, which facilitates rapid heat transfer to the surrounding area. On the other hand, the surface structure of a PSSP has a large number of furrows and protrusions, which creates conditions sufficient for multiple reflections of the light until it is absorbed, and combined with the light-absorbing effect of CNT, this structural aspect can facilitate a quite strong photothermal performance. Due to the fact that the 5% sample exhibits better CNT networking than does the 3% (which has a poor network due to having less CNT content) and the 7% (which has a poor network due to its increased agglomeration and voids), the 5% CNT-PSSP system exhibits a performance which is more favorable for absorbing light and converting it into heat, compared to the other two. This provides the conditions for the 5% CNT-doped PSSP to have quite superior photothermal performance.

### 3.5. Mechanical Stability

In order to study the effects of the long-term use of the material, a running-water scouring experiment of up to 5 min was carried out on the material. The surfaces of the three materials with different doping ratios were washed with flowing water at a flow rate of 22.5 mL/s, after which the contact angles of the three materials were tested. The process of this experiment is illustrated in support information and Figure S2.

**Figure 8:**
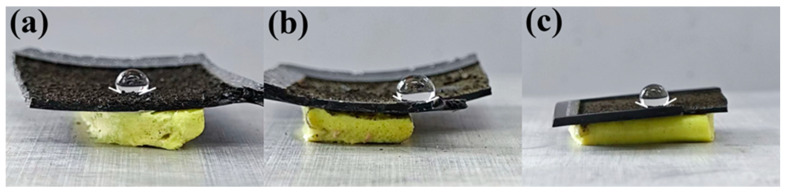
The superhydrophobic behaviors of the three samples after the water washout experiment; (**a**), (**b**), and (**c**) were 3%, 5%, and 7% CNT-doped PSSP samples, respectively.

As shown in Figure 8, the results show that the CA of all three samples are still above 145 degrees, after considering the measurement error, which indicates that all three samples have good mechanical stability and can be used in complex environments for a long time.

## 4. Conclusions

In this work, three patterned superhydrophobic photothermal materials (PSSP) with different CNT-doping ratios were successfully fabricated by simple laser-processing modification methods. All three materials exhibited excellent shape-memory performance, with nearly a 100% recovery rate. The 3% CNT-doped PSSP nanocomposite system demonstrated enhanced anti-icing efficacy, as evidenced by a prolonged ice-accretion duration of 485 s under −20 °C conditions. The material represented by 5% CNT-doped PSSP showed excellent photothermal performance; the de-icing took only about 30 s, and the surface of the material reached a high temperature of 180 °C within one minute. Persistent self-cleaning surface characteristics qualify this material for exterior applications demanding maintenance-free operation. The dual-action ice suppression mechanism—combining dust repellency via superhydrophobicity with dynamic photothermal-responsive de-icing through shape-memory actuation—establishes this material as a promising candidate for cryogenic infrastructure protection in extremely cold environments.

## Data Availability

The original contributions presented in this study are included in the article/Supplementary Materials. Further inquiries can be directed to the corresponding authors.

## Supplementary Materials

The following supporting information can be downloaded at: https://www.mdpi.com/article/10.3390/ma18112540/s1. Figure S1: Experimental apparatus for photothermal performance; Figure S2: Experimental apparatus for abrasion resistance; Video S1: 3% CNT shape memory performance; Video S2: 5% CNT shape memory performance; Video S3: 7% CNT shape memory performance; Video S4: 3% CNT self-cleaning performance; Video S5: 5% CNT self-cleaning performance; Video S6: 7% CNT self-cleaning performance; Video S7: 3% CNT anti-icing performance; Video S8: 5% CNT anti-icing performance; Video S9:7% CNT anti-icing performance.
