# Supplementary material for: Design of a Superhydrophobic Photothermal Shape-Memory Material Based on Carbon-Nanotubes-Doped Resin for Anti-Icing/De-Icing Applications
Source: Materials (Basel). 2025 May 28;18(11):2540. doi: 10.3390/ma18112540 (PMC12156130; doi:10.3390/ma18112540)
Supplement: Supplementary file 1 [file materials-18-02540-s001.zip › Supporting info.docx]

**Design of a Superhydrophobic Photothermal Shape-Memory Material Based on CNT-Doped Resin for Anti-Icing/De-Icing Applications**

In order to study the photothermal properties of materials, an experimental setup was built. The material is used to stand on the surface of the square steel with adhesive tape, the thermocouple is fixed on the anti-icing surface of the material, the laser with a wavelength of 808nm and a power of 1W is fixed above it for irradiation, and the light source is switched on and off for a cycle of 1min, during which the thermocouple is used to measure the surface temperature data of the material.

In order to study its photothermal de-icing performance, a drop of water was added to the surface of the thermocouple, and the square steel with the material and the thermocouple was placed in a refrigerator at -15 °C to wait for it to freeze.


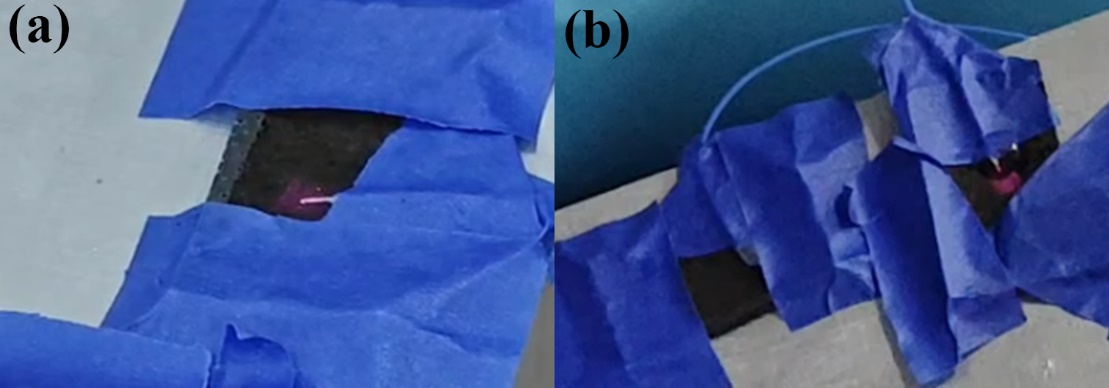


Figure S1 (a) Photothermal cycling test facility (b) Photothermal de-icing facility

In order to study the effect of long-term use on the material, a running water scouring experiment of up to 5 minutes was carried out on the material. The surfaces of three materials with different doping ratios were washed with flowing water at a flow rate of 22.5 mL/s, after
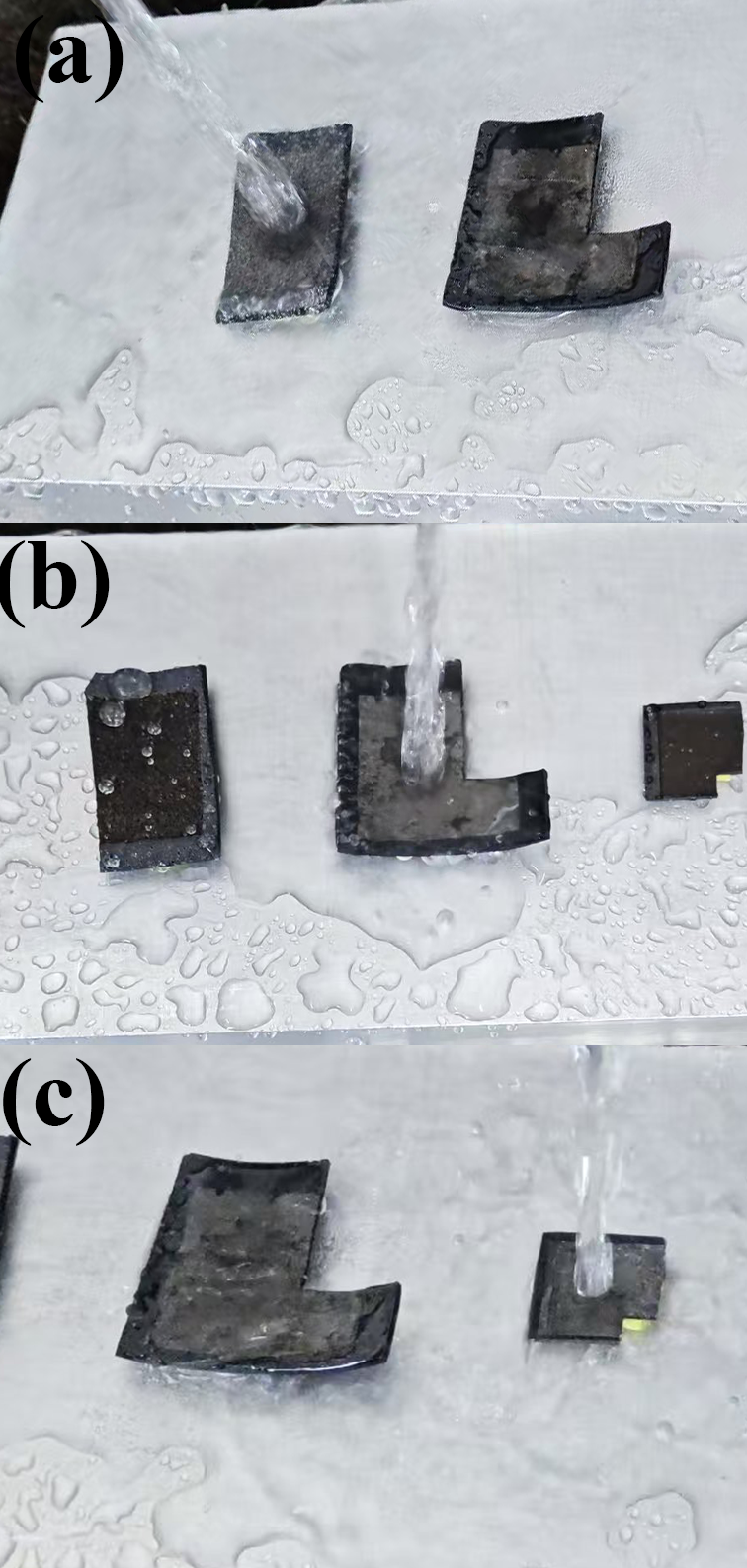
which the contact angles of the three materials were tested.

Figure S2 Three samples were washed using a water stream,(a), (b) and (c) are samples with CNT doping concentrations of 3%, 5% and 7%, respectively.

In the experiment, we heated the samples with three doping ratios to 70°C to a tem-perature above Tg, bent them into a "U" shape (0s state) and cooled them, and then placed them on a laboratory bench heated to 70 °C for shape restoration experiments and took videos to observe the relationship between their recovery state and time. Videos of 3%, 5%, and 7% CNT-doped materials were Video S1, Video S2, and Video S3, respectively.

The self-cleaning test was carried out on three samples, Quartz sand particles were deposited onto the substrate surface to simulate particulate contamination, followed by 50μL water droplet rinsing which effectively removed the adhered pollutants through self-cleaning action inherent to the superhydrophobic interface. Videos of 3%, 5%, and 7% CNT-doped materials were Video S4, Video S5, and Video S6, respectively.

Under subzero ambient conditions (-20°C), water droplets deposited on the PSSP surface maintain optical transparency at the onset of exposure (t = 0 s),and become non-transparent after completely frozen. Videos of 3%, 5%, and 7% CNT-doped materials were Video S7, Video S8, and Video S9, respectively.
